# In Memoriam—Dr. Edward J. Whitney

**Published:** 1895-11

**Authors:** 


### IN MEMORIAM—DR. EDWARD J. WHITNEY.

At a meeting of the staff of the Brooklyn Hahnemann Hospital, held Monday, October 7th, Dr. R. K. Valentine was elected President. Resolutions of sympathy were adopted on the death of Dr. Edward J. Whitney, the former President of the Board. The resolutions were offered in the regular form, as follows:

"*Resolved,* That we enter upon our minutes a note of our profound sense of the loss incurred in the death of our late President, Dr. E. J. Whitney. As a friend, as a physician, as an officer of our staff we shall miss and mourn him. The relationship we all had with Dr. Whitney was so intimate and so long continued, the affectionate regard in which we held him was so brotherly, we feel that formal resolutions of regret and condolence would but ill express the sense of our great personal loss and of our sympathy with his family and intimate friends; but desiring to testify our respect and love for him, we, the members of this staff, hereby constitute ourselves a committee to establish a Whitney memorial bed in the Hahnemann Hospital, endowing it in perpetuity as our monument to one whose death we feel was but a translation to a brighter and a better world."

